# The Effect of Parenthood on Health Lifestyles: A Longitudinal Analysis Using Data from the German Socio-Economic Panel

**DOI:** 10.3390/ijerph23070926

**Published:** 2026-07-19

**Authors:** Maja Großbach, Philipp Linden, Nadine Reibling

**Affiliations:** 1 Faculty of Health Sciences, Fulda University of Applied Sciences, Leipziger Straße 123, 36037 Fulda, Germany; maja.grossbach@gw.hs-fulda.de; 2 School of Economic Disciplines, University of Siegen, Unteres Schloß 3, 57072 Siegen, Germany; philipp.linden@uni-siegen.de

**Keywords:** parenthood, health behaviors, health lifestyle index, longitudinal

## Abstract

**Highlights:**

**Public health relevance—How does this work relate to a public health issue?**

- Health behaviors are important determinants of health.
- The transition to parenthood is a critical life event during which health-related behaviors and daily routines often change.

**Public health significance—Why is this work of significance to public health?**

- This study provides evidence of a decline in healthy lifestyle scores following the transition to parenthood.
- Gendered patterns were observed, with mothers showing larger and more persistent declines in health lifestyle scores than fathers.

**Public health implications—What are the key implications or messages for practitioners, policy makers and/or researchers in public health?**

- Health promotion should support individuals—particularly mothers—during the transition to parenthood.

**Abstract:**

Parenthood has been linked to changes in health-related behavior, though studies have reported mixed findings on whether these changes are beneficial or harmful. To date, most research has focused on associations between parenthood and individual health behaviors, while research on the association with overall health lifestyles is limited. This study investigates how health lifestyles change following the transition to parenthood based on longitudinal data from the German Socio-Economic Panel (Sozioökonomisches Panel, SOEP) from 2008 to 2019. A Health Lifestyle Index (HLI) was constructed including the health behaviors physical activity, diet, smoking, and sleep. We use longitudinal group trend regression models to account for unobserved heterogeneity between parents and childless individuals. A decline in the HLI following childbirth was found, with the largest reduction observed in physical activity. For women, the decline in HLI scores was more pronounced than for men. Over time, the decline in HLI levels stagnated and did not revert to pre-birth levels during the 5+ years post-birth observed in this study. These results indicate that the transition to parenthood is associated with a shift towards a less healthy lifestyle. Women were disproportionally affected, highlighting gendered inequalities in changes in health lifestyles following the transition to parenthood.

## 1. Introduction

Health-related behaviors are widely recognized as major risk factors for morbidity and mortality and seen as key drivers of the expansion of non-communicable diseases worldwide [1]. It is increasingly acknowledged that these health-related behaviors are not primarily the result of individual choices, but reflect complex processes shaped by structural factors such as class, gender, and living circumstances [2,3,4]. While some structural factors are relatively stable, others—like living circumstances—change during critical life transitions. This study examines how health behaviors change following the transition to parenthood. Most longitudinal research has focused on single behaviors in isolation [5,6,7], with few exceptions [8,9,10,11], yet the combined effect across behaviors is crucial for health outcomes. Therefore, we analyze how health lifestyles change in women and men following the transition to parenthood, guided by Cockerham’s Health Lifestyle Theory [12]. This framework defines health lifestyles “as collective patterns of health-related behavior based on choices from options available to people according to their life chances” [12]. The theory emphasizes the interplay of structure and agency in shaping health lifestyles, drawing on Bourdieu’s concept of habitus [13] as the disposition to engage in health-related practices.

These class- and gender-shaped dispositions influence tendencies toward certain health-related practices, but do not determine individual behaviors. Living circumstances are also crucial for lifestyle choices. The transition to parenthood can create a challenge for maintaining a healthy lifestyle, because the arrival of a child substantially changes parents’ existing routines and requires additional time as well as energy [14]. Financial pressures might increase as well with the transition to parenthood, especially if a parent takes parental leave or reduces working hours [15]. Moreover, parenthood is tied to strong normative expectations around parenting, which can create stress for new parents [16]. These pressures may be particularly pronounced for mothers, as they still carry most of the caregiving responsibilities [14,17]. At the same time, parenting can also activate new resources and motivations towards a healthy lifestyle. Many (expectant) parents report a heightened sense of purpose and motivation to make healthier decisions as they take on these new responsibilities [18,19].

The association between parenthood and individual health behaviors has already been explored in the existing literature. Most studies have found a decline in physical activity after the birth of the first child, which seems to be pronounced more strongly in mothers compared to fathers [5,20,21]. Research has also shown that new parents, especially during the first weeks postpartum, experience reduced sleep duration and quality, along with increased waking times after initially falling asleep [7,22]. A less clear trend has been observed regarding a change in nutritional behavior following the first birth. Some research implies a trend towards a healthier diet after transitioning to parenthood [23,24], whereas other studies suggest the opposite or no changes at all [24,25]. Research also suggests that, though many smoking mothers quit smoking during pregnancy, they tend to revert to pre-pregnancy smoking behaviors over time after becoming mothers [6,26]. Recent longitudinal studies further reveal differential effects of parenthood on mothers and fathers. Notably, women demonstrate greater and more persistent changes across several health behaviors compared to men [20,27,28].

Thus, overall, the existing research literature suggests that health behaviors may change differently following the transition to parenthood. However, as Cockerham [12] argues, health behaviors typically do not occur in isolation but rather as clustered patterns of practices, forming a health lifestyle. Analyzing changes in said health lifestyles, rather than isolated behaviors, allows the identification of overall changes in health behavior patterns, as individual health behaviors may compensate for or reinforce each other.

Only very few studies have studied the associations between parenthood and combined measures of health behaviors. Of those who did, two studies found a decrease in healthy behaviors [8,10], while one study found no significant effects [9] and one found a slight change towards healthier behaviors [11]. However, these few studies have important limitations. First, the studies focus exclusively on women, while only one study also looked at men [8]. Second, all four studies focus on specific subgroups rather than the general adult population. Bennett et al. compared women with and without Gestational Diabetes Mellitus [10]. Everett’s study focused exclusively on women who identify as lesbian [11]. Grace et al. conducted their study on healthcare workers from three different hospitals [9], and in Frech’s study, 90% of participants were under the age of 18 years at the first survey wave [8]. Consequently, these results cannot be generalized to the overall adult population. Finally, two studies compare parents’ outcomes to those of childless individuals, while the other two use parents’ observations before birth as a reference. However, none of the four studies combine within-person and between-person comparison and thus cannot distinguish changes associated with parenthood from general age-related changes or selection effects.

Given the limited research on the effects of parenthood on composite health behavior measures, along with the variability and methodological limitations of existing studies, this study aims to address this gap. Accordingly, our study addresses the following research question: Is the transition to parenthood systematically associated with changes in health lifestyles, and if so, to what extent do health lifestyles change over time following the onset of parenthood? In addition, we examine whether these trajectories differ between mothers and fathers. Health lifestyles will be measured using a Health Lifestyle Index (HLI) that encompasses physical activity, diet, smoking status, and sleep. Drawing on Cockerham’s Health Lifestyle Theory [12], for theoretical guidance, this work seeks to advance our understanding of these associations, changes over time, and their underlying mechanisms. This study employs longitudinal data from the German Socio-Economic Panel from 2008 to 2019 in a group trend regression model that compares first-time parents’ observations with those of childless individuals and pre-birth parents. Separate models are estimated for mothers and fathers to account for potential gender differences.

## 2. Theoretical Background

In his Health Lifestyle Theory, Cockerham argues that the interaction of social structures and individual agency produces health lifestyles [12]. He defines health lifestyles as sets of practices regarding health, for example, alcohol consumption or dietary habits. According to Cockerham, structural conditions, such as class, gender, and living conditions, shape individuals’ life chances, which define the probabilities of achieving their needs and desires. Life chances provide the context in which life choices, or individual agency, can take place. Together, structure and agency interact to determine the courses of action regarding health lifestyles through the habitus [12]. Originally conceptualized by Bourdieu, the habitus can be defined as a set of dispositions that determines how an individual perceives and interprets the social world and guides their behavior in daily life [13]. Resulting from these dispositions are the practices that influence health, such as diet, exercise, smoking, and alcohol consumption, which together build an individual’s health lifestyle. Both Bourdieu and Cockerham emphasize that these practices are mostly not part of conscious considerations but embedded in and maintained by the routines of daily life [12,13].

After the transition to parenthood, daily life shifts to accommodate the needs of the new child, especially during the newborn phase, where infants need constant care. Parents, therefore, experience a disruption in the routines that were part of their daily lives before childbirth. Routines, according to Bourdieu and Cockerham, are the main driver of the habitus, and consequently health behaviors, which is why parenthood is expected to affect health lifestyles [12,13].

Beyond the disruption in routine, which destabilizes the habitus, the transition to parenthood also has implications for parents’ life chances. For example, parenthood can create or reinforce financial pressures through child-related expenses or a decrease in income due to parental leave or a reduction in working hours [29]. Additionally, children’s constant need for care and supervision consumes much of the parents’ time and energy, especially during the first years of their lives [30]. Apart from these changes in resources, the role of a parent is tied to norms and expectations that could either motivate healthy decisions or act as a source of financial, emotional, or physical strain [16,31].

However, these effects are not experienced equally by both parents. Research shows that the transition to parenthood is associated with more pronounced and longer-lasting changes in health behaviors for women than for men [5,6,32]. For women, pregnancy, childbirth, and the postpartum period are physically and emotionally challenging [33]. Additionally, they carry most of the responsibility of caring for the child—not only during the postpartum period but also in the following years [17,29]. Consequently, women are still much more likely than men to take parental leave, reduce working hours, or leave employment after transitioning to parenthood [17,29,34], which increases their risk of financial distress. Furthermore, women face higher expectations and social pressures regarding their parenting qualities compared to men [35]. This subjects mothers to greater emotional strain and stress than fathers [30]. According to the Health Lifestyle Theory, these structural constraints shape mothers’ life chances by limiting time, financial resources, and autonomy available for health-promoting behaviors [12]. At the same time, gendered social norms and caregiving expectations become incorporated into the gender habitus, reinforcing patterns in which children’s needs are prioritized over the mother’s own health. For instance, mothers report that taking time to exercise is associated with feelings of guilt [36]. This gendered habitus is also reflected in the distribution of care responsibilities. Mothers often take on a larger share of time-sensitive and inflexible caregiving tasks, which may limit opportunities for health-promoting behaviors. For example, (breast-)feeding may affect sleep, while tasks such as cooking or putting children to bed may reduce the time available for physical activity [37].

Overall, these structural implications of parenthood show how parenthood can influence parents’ health lifestyles by affecting life chances and the routine that upholds the habitus. However, health lifestyles are not only dependent on structure and routine but also on agency, which parenthood might influence as well.

During the transition to parenthood, the parents’ life choices seem to become focused on the child’s wellbeing. Not only during pregnancy but also in later years, parents report avoiding engaging in unhealthy behaviors in the presence of their children [18,38]. They also seem to prioritize health behaviors that are aimed at the health of the family over those that are beneficial solely for their own health [18,39]. Besides ensuring their children’s wellbeing, parents also feel the responsibility of acting as a role model in the development of their children’s health lifestyles [18,19]. These intentions reflect parents’ awareness of their role in the socialization process, which Cockerham [12,40] views as a major contributor to the development of the habitus and health practices.

Overall, these examples demonstrate that parenthood can influence health behaviors in different directions. Both Bourdieu [13] and Cockerham [12] conceptualize (health) lifestyles as internally consistent, suggesting that they gravitate to either healthier or less healthy patterns of behavior. However, this section showed that parenthood may lead to healthier behaviors in some domains and less healthy behaviors in others. It is therefore necessary to examine the overall net effect of parenthood on health lifestyles. It must also be determined whether potential changes in health lifestyles are limited to a certain time after childbirth or if they are more permanent. The former could suggest that changes are mostly the result of a disruption in routine, while the latter could imply a fundamentally changed habitus. Both cases have been observed in previous research [7,32,41].

## 3. Materials and Methods

### 3.1. Data

This study uses data from the German Socio-Economic Panel (SOEP, Version 36, [42]), a representative longitudinal study located at the German Institute for Economic Research (DIW Berlin). Since 1984, around 30.000 individuals and 15.000 households have participated in annual questionnaire-based interviews. The main topics covered include demographics, socioeconomics, housing, health, and questions on personal values and attitudes [42].

The harmonized raw dataset included a total of *N* = 346,771 observations. The observation period for this study was from 2008 to 2019. For the analyses, the sample was restricted to women and men who were at least 18 years old and born between 1960 and 1991, thus representing birth cohorts most likely to become first-time parents during the observation period [43,44]. Parents were only included if they were observed at least once before childbirth so that within-person changes over the course of transitioning into parenthood could be observed. Furthermore, respondents were excluded if they had any missing values in the control variables.

Due to different data collection patterns for each health behavior variable (see Table 1), 2008 was the only year in which all four health behaviors were collected simultaneously, leaving the years 2009–2019 with incomplete data for the computation of the HLI. To still be able to compute the HLI, the years 2009–2010, 2011–2012, 2013–2014, 2015–2016, and 2017–2018 were combined into two-year periods, respectively. Afterwards, a total 15.33% of values were still missing across the four health behavior variables. To eliminate the remaining fraction of missing values, multiple imputation (MI) was conducted, using predictive mean matching and performing *m* = 20 imputations as proposed by White et al. [45]. The imputation was executed in Stata (version 16.1) using the mi command [46]. Robustness checks revealed no significant differences between the original and imputed data (the results are available upon request from the authors).

In total, the sample included *N* = 25,692 observations from *N* = 7049 observed individuals (3287 women and 3762 men), of whom 791 transitioned to parenthood during that time (429 motherhood and 362 fatherhood).

### 3.2. Health Lifestyle Index Construction

In the existing literature, health lifestyles are commonly measured either by validated health lifestyle questionnaires [47] or by a simple sum score of four to five health behavior variables [48]. As this study uses secondary data, the former approach was not possible. An overview of research using the latter approach is provided in Appendix A. HLIs most often include measures of physical activity, diet, alcohol consumption, smoking behavior, and Body Mass Index (BMI). In this study, alcohol consumption was excluded because it was only measured at two time points during the SOEP data collection since 1984, providing insufficient data for the effect analyses. BMI was not included in the index because research suggests that it has limited explanatory value with respect to a person’s overall health due to its inability to account for factors such as age, ethnicity, and body composition [49]. In addition to physical activity, nutrition, and smoking habits, we also use sleep as a health-related behavior. Current research associates both sleep duration and sleep satisfaction with physical and mental health outcomes [50,51]. Previous work has shown a decline in both measures after the transition to parenthood [7], making sleep an important health behavior in parental health lifestyles.

Thus, the primary measure used in this study is an HLI that we constructed as a composite measure of the four health behaviors: physical activity, diet, smoking, and sleep. Each variable was included with the same weight and therefore recoded into three categories, ranging from 0 for poor healthy behavior to 2 for healthy behavior (see also Appendix B for an overview). Using the imputed data, the values for all four health behaviors were then summed for each participant and wave, constructing the HLI for all 20 imputations. The final HLI ranges from 0 for the least healthy lifestyle to 8 for the healthiest lifestyle, and it was treated as a metric variable for further analyses.

### 3.3. Health Behavior Variables

For *physical activity*, participants were asked to state how frequently they participated in sports. The World Health Organization recommends at least 150 min of moderate or 75 min of vigorous physical activity per week for adults [52]. Following these recommendations, the answers were categorized into 0 = ‘never’ [never], 1 = ‘irregularly’ [every month/less frequently], and 2 = ‘regularly’ [every day/every week].

Participants’ *diet* was measured by asking to what extent they follow a health-conscious diet. Responses were recoded into 0 = ‘not at all’ [not at all], 1 = ‘a little’ [a little], and 2 = ‘strong’ [very strong/strong]. This measure reflects participants’ subjective perception of healthy eating rather than compliance with established dietary guidelines.

For *smoking*, four survey items were combined to determine participants’ current smoking behavior. First, participants were asked whether they currently smoke [yes/no]. Those who reported being current smokers were then asked to state the number of cigarettes, pipes, and cigars consumed in a typical day. These numbers were then categorized into two categories: 1 = ‘light smokers’ and 2 = ‘heavy smokers’. Cigarette smokers were classified as heavy smokers if they reported smoking at least 20 cigarettes per day. This number equals approximately one pack of cigarettes and is commonly used as a threshold to distinguish between light and heavy smokers in the scientific literature [53,54,55]. The number of pipes and cigars was categorized using cigarette equivalents proposed by the Robert Koch-Institute, Germany’s public health institute [56]. According to their recommendations, on average, one cigarette contains one gram, one cigar contains four grams, and one pipe contains three grams of tobacco [56]. Using these equivalents, the threshold for heavy smokers was set for at least seven pipes or five cigars per day. Smoking behavior was then categorized into the following: 0 = ‘light smoker in at least two categories or heavy smoker in at least one category’, 1 = ‘light smoker in one category and nonsmoker in the other two’, and 2 = ‘nonsmokers’.

For *sleep*, participants were asked to state their average sleep duration in hours for normal workdays, as well as satisfaction with their sleep on a scale from 0 = ‘completely dissatisfied’ to 10 = ‘completely satisfied’. Sleep duration and sleep satisfaction were combined into a single measure, as previous research has shown that both sleep duration and sleep satisfaction are associated with physical and mental health outcomes [50,51]. In line with scientific evidence for recommended sleep durations in adults [57,58], sleep duration was categorized into ‘sufficient’ [6–9 h] and ‘insufficient’ [<6 or >9 h]. Satisfaction with sleep was categorized into ‘satisfied’, including values from 6 to 10 on the satisfaction scale, and ‘not satisfied’ for values from 0 to 5. The final categorization of the sleep variable was 2 = ‘sufficient and satisfied’, 1 = ‘insufficient or unsatisfied’, and 0 = ‘insufficient and unsatisfied’.

### 3.4. Parenthood

The central predictor used in this study is the transition to parenthood, meaning the birth of the first child. Parenthood itself was thereby operationalized in two different ways: time-sensitive and time-constant. The *time-sensitive* parenthood variable indicates the years before and after the transition to parenthood. It was categorized into the following: more than three years before birth (‘BY−3a+’), more than one to three years before birth (‘BY−1–3a’), one year before to one year after birth (‘BY−/+1a’), more than one to three years after birth (‘BY+1–3a’), more than three to five years after birth (‘BY+3–5a’), and more than five years after birth (‘BY+5a+’). The category ‘BY−3a+’ was also used to include individuals who did not report having children during the observation period and was thus used as the reference category in the analyses. The *time-constant* parenthood variable indicates whether an individual has ever been observed as a parent during the observation period. Its values are (0) for never being observed as a parent and (1) for being observed as a parent at least once. Both parenthood variables were created using birth history data on participants’ children.

### 3.5. Control Variables

Sociodemographic data used for the control variables included participants’ age, birth cohort (‘1960–1969’/‘1970–1979’/‘1980–1991’), partner status at birth (‘no partner’/‘partner’), migration background (‘none’/‘direct or indirect’), survey years, and education level at birth. Education was measured using the Comparative Analysis of Mobility in Industrial Nations (CASMIN) scale. Education levels were dichotomized into ‘primary/secondary’ and ‘tertiary’.

### 3.6. Statistical Analysis

This study aims to estimate the effect of parenthood on parents’ health lifestyle (*Y*). The causal parameter of interest here is the Average Treatment Effect on the Treated (*ATT*), which compares parents’ observed health lifestyles with the hypothetical health lifestyles they would have experienced had they remained childless [59,60]. The treatment is defined here as an individual’s parenthood status, with *X* = 1 denoting parenthood and *X* = 0 denoting childlessness within the observed timeframe. The *ATT* can be expressed as follows:

(1) $$ATT=P\left[Y_{x=1}|X=1\right]-P\left[Y_{x=1}|X=0\right].$$

The *ATT* can be split into two components, the first being parents’ *HLI* using observations on parents across time, controlled for specific control variables *Z*, while the second mirrors the same *HLI* values but under the condition of not having become parents. The second part is therefore counterfactual and not observable [60]. Estimating the *ATT* therefore requires assumptions that allow the missing counterfactual to be estimated using observed data.

The first assumption is that, apart from parenthood, there are no unmeasured differences between parents and childless individuals affecting the outcome. Under this assumption, a regression model with control variables *z* would yield an unbiased estimate of the *ATT*. In practice, however, this is unlikely to work as parents and childless individuals may differ in unobserved characteristics such as their physical and mental constitution.

A second assumption is the parallel trend assumption, which expects such differences to exist but requires both groups to follow parallel trends before treatment. This may be captured by adding a time-constant parenthood indicator, which would extend the model to a difference-in-difference approach. However, because parents and childless individuals may already differ in their health lifestyle trajectories before the transition to parenthood, the parallel trend assumption may not hold. Consequently, standard DiD estimates may remain biased.

To address this, the third assumption is that parents’ health lifestyles would have followed their observed pre-birth trend had they not become parents. Under this assumption, any observed deviation from that trend can be interpreted as causal effects of parenthood and thus the *ATT*. We therefore estimate a pooled OLS group trend model (POLS-GT) by incorporating an interaction term between the time-constant parenthood indicator and age, leading to the following regression formula:

(2) $$P\left[Y_{t}|X_{t},Z_{k}\right]=\theta_{0}+\theta_{1}x_{t}+\theta_{2}x+\theta_{3}{xa}_{t}+\theta_{k}\;z_{k}.$$

The POLS-GT model therefore accounts for both differing trends in the control variables *z* and the time-constant differences between parents and childless individuals [61]. If the trend is modelled correctly, the coefficient *θ*_1_ can be interpreted as the *ATT*. We run the model for both the *HLI* and individual health behavior variables to model the overall trend and to visualize the influence of individual behaviors.

All models were estimated separately for men and women to account for potential gender differences. The analyses were performed using Stata version 16.1 [62]. For all statistical tests, we used a significance level of *α* = 0.05, which is conventionally used in social and health sciences [63]. We did not apply formal corrections for multiple comparisons, since our outcomes and time points are not measured independently [64]. A correction would risk the inflation of Type-II errors [65,66].

## 4. Results

### 4.1. Descriptive Results

This study’s main objective was to find out whether the HLI differs between first-time parents and both their pre-birth observations and childless individuals, indicating an effect of parenthood on individuals’ health lifestyles. Table 2 shows the characteristics of the analysis sample for childless individuals and parents before and after their first birth.

The proportions of female respondents were higher in the pre- (54.3%) and post-birth (55.4%) groups compared to the childless group (44.9%). Pre-birth parents had the lowest mean age (28.7 ± 5.4 years), while post-birth parents had the highest mean age (34.8 ± 5.9 years). A notable difference can be seen in educational status. Approximately half of the parents reported tertiary education levels compared to 30.1% among childless individuals. Similarly, partner status shows a strong contrast across groups, with over 97% of parents reporting living with a partner, whereas the majority of childless individuals do not report having a partner (59.9%). Regarding migration background, only minor differences were observed across the groups. The majority of respondents in all three groups were born between 1980 and 1991, with the largest proportion observed in pre-birth parents (69.2%). Only around 3% of both the pre- and post-birth groups were born between 1960 and 1969 compared to 25.4% of childless individuals. Finally, the mean HLI scores are relatively similar across the groups, with the highest value observed among pre-birth parents (6.0 ± 1.5), followed by post-birth parents (5.8 ± 1.5) and then childless individuals (5.7 ± 1.6).

### 4.2. The Effect of Parenthood on Health Lifestyle

**Figure 1:**
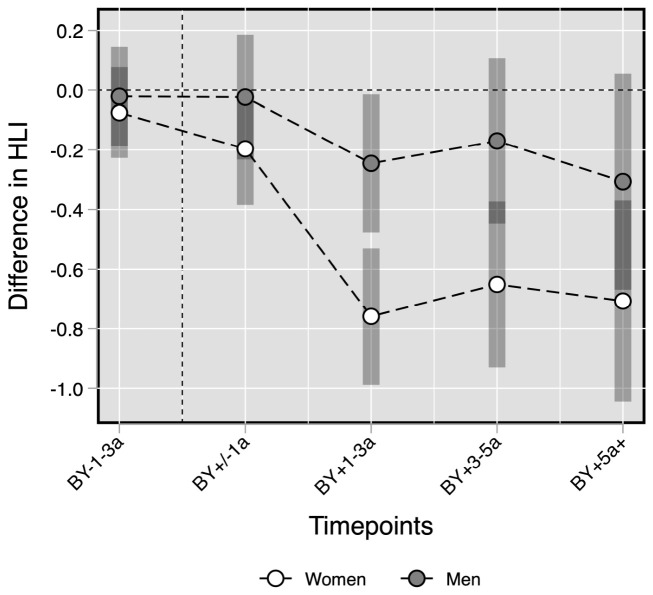
Difference in HLI over time between parents and childless individuals. Source: Own compilation and depiction using SOEP data, *N* = 25,692 person-years. Point estimates from pooled OLS group trend models stratified by gender, controlling for age, educational level, relationship status, migration status, survey year, birth cohort, and the interaction between the parenthood indicator and age, with 95% confidence intervals based on robust standard errors. The dashed horizontal line shows the zero difference; the dashed vertical line shows the time of the transition to parenthood.

Figure 1 shows the estimated difference in overall HLI score over time for women and men compared to the reference category (BY−3a+) from the pooled OLS group trends model. For women, the HLI is significantly lower following the birth of the first child compared with childless women and mothers more than three years before birth. The largest difference is observed one to three years after birth with −0.8 on the 0–8 HLI scale, representing roughly 10% of the total scale range (*b* = −0.759, *p* < 0.01). The estimates in the following years are comparable, suggesting that lower HLI scores among women persist for five years or longer. For men, the observed reduction in HLI is smaller and only significant one to three years after the birth of the child.

### 4.3. The Effect of Parenthood on Health Behaviors

**Figure 2:**
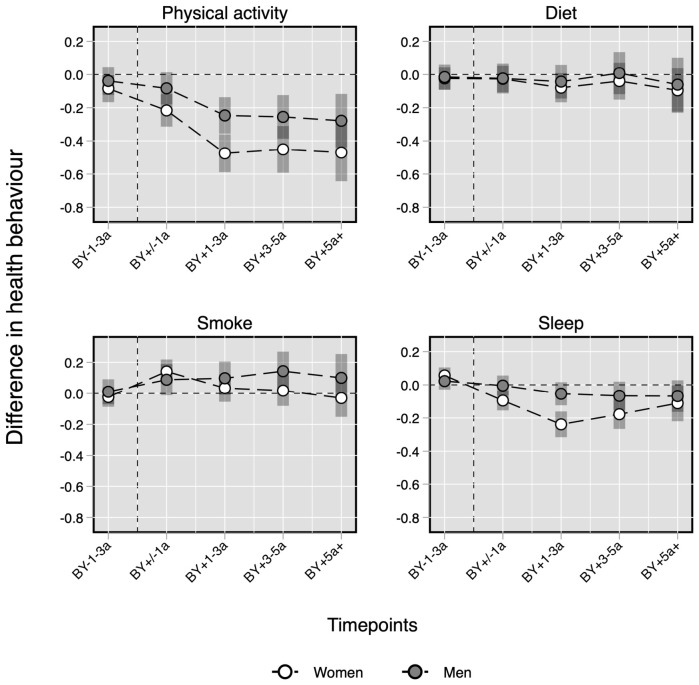
Differences in health behaviors over time between parents and childless individuals. Source: Own compilation and depiction using SOEP data (v.36), *N* = 25,692 person-years. Point estimates from pooled OLS group trend models regressing the individual health behavior variables stratified by gender, controlling for age, educational level, relationship status, migration status, survey year, birth cohort, and the interaction between the parenthood indicator and age, with 95% confidence intervals based on robust standard errors. The dashed horizontal line shows the zero difference; the dashed vertical line shows the time of the transition to parenthood.

Figure 2 shows the differences in the four health behaviors over time between parents and childless individuals compared to the reference category (BY−3a+) from separate pooled OLS group trend models for each behavior. The regression tables for each model can be found in Appendix C.

For both men and women, *physical activity* levels were lower following the birth of the first child than in the reference category. For women, this decline is stronger, reaching the maximum difference between mothers and childless women more than one to three years after birth (−0.475, *p* < 0.01) and recovering slightly afterwards. The effect was significant at every time point. Among men, physical activity levels remained lower over a longer period, although the decline was less pronounced, reaching the maximum difference more than five years after birth (−0.278, *p* < 0.01). Here, the effects were significant at every time point starting more than one to three years after birth. This represented the largest change among the four health behaviors, suggesting that differences in physical activity account for most of the observed decline in the overall HLI. A slight decline in a healthy *diet* compared to the reference category can be observed over time in both genders. Women’s diet scores declined almost steadily after birth, reaching the maximum difference more than five years after birth (−0.095, *p* = 0.171). In men, a similar trend in declining diet scores has been observed, peaking more than five years after birth (*b* = −0.061, *p* = 0.467). Although diet scores tended to decline for both women and men after childbirth, the estimated effects were small and non-significant, suggesting only limited evidence for meaningful dietary changes. Regarding *smoking* behavior, coefficients were generally positive for both women and men, indicating a tendency toward lower smoking propensity following the transition to parenthood. For women, the effect was the highest one year before to one year after birth (0.141, *p* < 0.01) and declined slightly after that. Apart from this time point, the effects were not significant for women. For men, the effects increased later, peaking more than three to five years after birth (0.143, *p* < 0.05) and declining slightly after that. The effects were not significant at any other time point. While the coefficients indicate a tendency towards reduced smoking after parenthood, these effects were generally small and statistically inconsistent in both genders. Women had lower *sleep* scores at every time point after birth, reaching the maximum difference more than one to three years after birth (−0.238, *p* < 0.01). Afterwards, the differences compared to the reference category become smaller again. The decline in sleep among women was smaller than the decline in physical activity but remained statistically significant across all post-birth periods, suggesting persistently less favorable sleep outcomes among mothers. Men’s sleep is only slightly worse than that of childless men and fathers more than three years before the birth. The maximum decline can be seen more than five years after birth (−0.068, *p* = 0.164). However, the effects for men were not significant at any time point.

Among the four health behaviors, differences in physical activity accounted for the largest share of the observed decline in the overall HLI, followed by sleep among women. Changes in diet and smoking were comparatively small.

## 5. Discussion

The aim of this study was to investigate how the transition to parenthood affects parents’ overall health lifestyles. Prior research using composite health indices has been scarce and has shown mixed results [8,9,10,11]. Moreover, existing studies were not representative of a broader adult population and did not address selection effects. This study extended previous research using longitudinal data from the German SOEP and pooled OLS group trend models that address time-constant and time-varying unobserved heterogeneity.

### 5.1. Main Findings

This study’s findings regarding changes in health behaviors following the transition to parenthood largely align with current research. For example, physical activity levels decreased significantly after the transition to parenthood for both genders, consistent with patterns observed in previous work [5,20,21]. Of all variables included in our analyses, physical activity showed the greatest and most long-lasting change after transitioning to parenthood in both women and men. These results indicate that the transition to parenthood presents a considerable challenge to maintaining physical activity habits.

Healthy diet scores declined slightly in both genders with the transition to parenthood, though not significantly. In current research, there are mixed findings on changes in diet after transitioning to parenthood [24,67]. However, the findings regarding diet in this study may be biased due to the subjective nature of the question assessing the extent to which participants follow a health-conscious diet.

A reduction in smoking propensity was found in both genders after the first birth. For men, the reduction in smoking propensity extended until five years after birth, after which it increased again. For women, smoking propensity was only lower one year before and one year after birth, and it increased again thereafter. However, the time-varying effects were only significant one year before to one year after birth for women and more than three to five years after birth for men. Everett et al. found no significant differences in smoking between mothers and childless women [11]. Other studies found that the majority of women quit smoking during pregnancy [6,26]. In the years after pregnancy, some women resume smoking, while others quit permanently [6,26]. This study’s findings could imply similar patterns, as the category ‘one year before to one year after birth’ covers interviews carried out anytime between pregnancy and one year postpartum. Thus, the observed trend regarding smoking behavior among mothers is consistent with previous findings that many women quit smoking during pregnancy, potentially due to increased health awareness and concerns related to the unborn child, and after birth, over time, some mothers start smoking again. This illustrates that the transition to parenthood may also be accompanied by positive changes in some health behaviors. Increased health awareness and a stronger sense of responsibility for the child’s well-being may motivate parents to adopt health-promoting practices, even if these positive changes are not equally evident across all health behaviors. A cross-sectional study of a large representative German sample found no difference in smoking prevalence between childless individuals and parents of one child but lower smoking prevalence in parents with two or more children [68]. Contrary to our findings, this study further reported higher rates of physical activity in mothers and healthier diets in parents compared to childless individuals. These differences may be due to the study’s cross-sectional design, which does not capture within-individual changes over time [68].

Regarding sleep patterns, this study found a significant decline in sleep duration and satisfaction in women after birth. Sleep levels increase again, starting more than three years after birth. Men’s sleep levels declined slightly over time, though not significantly. Other studies on parents’ sleep show a pronounced decline in sleep duration and satisfaction after birth [7,69]. This effect often seems to be most pronounced in the first weeks postpartum and tends to revert over time, as babies surpass the stage of their life in which they need to be fed and cared for regularly throughout the night [7]. The findings are consistent with the interpretation that changes in sleep duration and quality among parents may be related to infants’ need for frequent care during the first months of their life.

Overall, lower HLI scores were observed following the transition to parenthood, which was the result of a strong and long-lasting decrease in physical activity, a slight and insignificant decrease in healthy diet, a slight decrease in unhealthy smoking behavior, and a decrease in—particularly mothers’—sleep levels. Thus, although some components of the HLI balance each other out, there is an overall decline in health lifestyle scores following the transition to parenthood. However, the observed reduction in smoking behavior demonstrates that the transition to parenthood may also create opportunities for positive health behavior change. This supports the notion that parenthood is not exclusively a period of health decline but rather a complex life transition in which competing demands and new motivations coexist. In our study, however, the observed declines in physical activity and sleep outweighed these positive behavioral changes, resulting in an overall decline in HLI.

The associations between parenthood and almost all health behaviors, as well as overall HLI scores, were more pronounced among women than among men. The largest difference in HLI compared to the reference category was observed more than one to three years after birth for women, with a reduction of almost −0.8 HLI score points. This effect represents more than 0.5 standard deviations of HLI in parents (SD = 1.5), which has been suggested as a benchmark for a relevant difference in health-related quality of life outcomes in the previous literature [70]. Also, women showed longer-lasting changes in overall health lifestyle than men. In the recent literature on gender differences in the effects of parenthood on health behaviors, similar trends have been observed, indicating that women’s health lifestyles appear to change more substantially following the transition to parenthood compared with men’s [5,6,20,27,28,32]. The stronger initial decline in health lifestyle after birth in women may partly reflect the physical and emotional strains associated with childbirth and the postpartum period [33]. Additionally, women still carry the majority of responsibilities concerning care work and housework and face different social expectations than their male partners [29,35]. Beyond the structural mechanisms discussed above, the transition to parenthood also fundamentally changes couple dynamics, which may contribute to changes in health-related behaviors. Parenthood often entails a renegotiation of household and caregiving responsibilities, changes in relationship quality, and increased parenting-related stress [71,72]. These changes may influence health behaviors both directly (e.g., through stress-related changes in sleep or health practices) and indirectly by shaping the support, resources, and opportunities available for maintaining healthy lifestyles. Future research should therefore consider couple-level processes alongside structural determinants to better understand the mechanisms linking parenthood and health lifestyles. Moreover, parenthood may influence additional dimensions of adult health and well-being, including sexual health and intimacy, which are closely related to relationship quality and psychological well-being [73]. While these aspects are beyond the scope of the present study, they represent important topics for future research on parental health.

From the perspective of Cockerham’s Health Lifestyle Theory, the findings suggest that the transition to parenthood constitutes a major life event that is associated with changes in individuals’ life chances and opportunities to engage in health-promoting practices. The observed decline in health lifestyles, particularly among mothers, illustrates how structural constraints such as caregiving responsibilities, reduced time resources, and gendered social expectations may limit individuals’ health-related choices despite existing intentions to maintain healthy behaviors [36]. In line with Bourdieu’s concept of habitus, these structural conditions become embedded in everyday routines and practices, contributing to persistent gender differences in health lifestyles beyond the postpartum period. Thus, the present findings support the assumption that health lifestyles are not solely the result of individual choice but are shaped by broader social and structural circumstances.

### 5.2. Limitations

While this study provides valuable insights, it is important to consider several limitations that may affect the interpretation of the findings. In particular, potential sources of bias can be seen in the reliance on self-reported health behavior measures, the limited operationalization of socioeconomic status, and the presence of missing data.

First, the variables included in the HLI were not specifically designed for this purpose and thus, in some cases, do not allow for an objective assessment of adherence to established health recommendations. For example, the diet variable (‘How much attention do you pay to maintaining a healthy diet?’) does not refer to specific criteria for a healthy diet. As a result, responses depend on the respondent’s interpretation of healthy eating rather than on scientifically based diet recommendations. Similarly, the physical activity variable does not allow for a detailed comparison of participants’ physical activity levels with recommendations by the WHO, as it does not include the amount and intensity of physical activity. All health behavior variables were also self-reported, which could lead to bias due to false recollection or social desirability. However, despite the limitations of the health-behavior variables for operationalizing a healthy lifestyle, they still provide a reasonable basis for an initial analysis of the parenthood effect in a longitudinal, representative sample.

Second, all health behavior variables were categorized into three groups, distinguishing between healthy, moderately healthy, and unhealthy behaviors. Though this is already a more nuanced distinction than seen in other studies in which health behaviors were dichotomized, it does not account for small-scale changes in health behavior. If, for example, a respondent would go from one cigarette per day to 19 cigarettes per day, the HLI would not be able to depict this change, even though there is a significant increase in daily cigarettes and associated health implications.

Third, this study did not fully account for socioeconomic status. It aimed to examine changes in health lifestyles due to parenthood through the perspective provided by Cockerham’s Health Lifestyle Theory, which builds on Bourdieu’s concept of habitus [12,13]. Both frameworks emphasize the influence of class circumstances in shaping the habitus and, consequently, health-related practices [12,13]. In the present study, however, socioeconomic status was only reflected through participants’ level of education. The reason is that the transition to parenthood often results in changes in employment and earnings, e.g., due to parental leave and part-time work, rendering these variables endogenous to the parenthood effect. Therefore, employment and income were not included as control variables. Although education is a good proxy for socioeconomic status or class position [74], a more comprehensive measure of socioeconomic status could have provided a more differential distinction of socioeconomic groups, especially considering the present theoretical framework.

Furthermore, a considerable limitation of this study lies in the proportions of missing values in the health behavior variables. To address this issue, the waves from 2009 to 2018 were combined into pairs of two years each, which greatly reduced the proportions of missing values from 37.88% to 16.68% among the dependent variables. However, the diet variable contained no observations in the last three waves (2015–2016, 2017–2018, and 2019). The remaining missing values were imputed using MI. The missingness mechanism is best characterized as Missing Completely at Random (MCAR), as the absence of diet data in the 2015–2019 waves resulted solely from the survey design (questions were not administered) and was independent of respondent characteristics or outcomes. Consequently, MI is the recommended strategy in this situation [75]. Robustness checks did not reveal significant differences between the original and imputed data, supporting the plausibility of our estimates.

Finally, the time-sensitive parenthood variable was not implemented as a continuous but as a categorical variable. This specification does not allow for an inspection of trajectories on a yearly basis, for example, between the beginning of pregnancy and after the postpartum period. However, it enables us to test for the parallel trend assumption between parents and childless individuals, even if the time of birth is “only” within a certain time window, which is the central aim of this paper and our model.

### 5.3. Implications and Future Research

The present findings have important implications for public health policy. They emphasize the need for preventive and health-promoting interventions that support expectant and first-time parents in maintaining and rebuilding healthy routines. The family is a key setting in which health behaviors are formed, making it a vital starting point for health promotion efforts. This is particularly relevant for parents, as health behaviors in midlife are associated with later-life health outcomes while also influencing the children’s developing health behaviors [76,77]. The positive trend observed in the decline of harmful smoking patterns indicates that the transition to parenthood may represent a window of opportunity for positive health behavior changes. This notion is supported by interventional studies targeting positive changes in health behaviors during pregnancy and early parenthood [78,79]. Interventions approaching the health behavior of (expecting) parents should aim at improving parents’ self-regulation skills and strengthening their social support [78,80]. As women showed more pronounced changes in health lifestyles following the transition to parenthood, interventions tend to be more successful for women than for men [78,79]. Nevertheless, these interventions should be carried out at a couple- or family-based level, as the involvement of both partners is an important factor for the interventions’ success [80,81]. In addition, structural measures such as affordable or subsidized childcare and child allowance may positively affect parental health through the reduction in economic burden and financial insecurity associated with parenthood.

These findings suggest that promoting healthy lifestyles during the transition to parenthood requires not only interventions on an individual level but also structural policies that reduce caregiving burdens, challenge existing care norms, and thereby facilitate the maintenance of healthy routines.

Future research should further investigate the association between parenthood and health behavior in women and men and the underlying structural and behavioral mechanisms, especially in longitudinal designs. As this study examined only a limited timeframe after childbirth, future research should explore whether effects persist or change in the longer term. When using an HLI, future studies should ideally include health variables that allow for a more differentiated categorization along evidence-based recommendations. Moreover, the structural conditions of parenthood are constructed by the cultural and institutional characteristics of countries [20]. Therefore, the effects of parenthood on health lifestyles can vary between countries, so future research should examine the effects of parenthood on health lifestyles using longitudinal data from other countries.

## 6. Conclusions

Overall, the findings highlight the challenges associated with maintaining healthy lifestyle practices during the transition to parenthood and underline the importance of daily routines and broader structural factors in shaping health lifestyles. The findings support Cockerham’s theoretical perspective, in which health lifestyles are not merely the result of autonomous decisions but are practices rooted in structural living conditions upheld by routine [12]. To address these challenges, health-promoting interventions should support (expectant) parents in maintaining healthy routines after childbirth, for example, through family-oriented and socially supportive intervention approaches aimed at improving parents’ self-regulation skills. Additionally, structural measures should aim at reducing parents’ economic burden. Further research is needed for a more differentiated analysis of the longitudinal effects of parenthood on health lifestyles and underlying structural and agentic mechanisms.

## Figures and Tables

**Table 1:** Data collection time points for health behavior variables.

	Physical Activity	Diet	Smoking	Sleep
2008	x	x	x	x
2009	x	-	-	x
2010	-	x	x	x
2011	x	-	-	x
2012	-	x	x	x
2013	x	-	-	x
2014	-	x	x	-
2015	x	-	-	x
2016	-	-	x	-
2017	x	-	-	x
2018	x	-	x	-
2019	x	-	-	x

Source: Own compilation and depiction using SOEP data. x = Variable was part of this year’s questionnaire. - = Variable was not part of this year’s questionnaire.

**Table 2:** Characteristics of individuals who are always childless, pre-birth parents, and post-birth parents.

	Always Childless (*N* = 19,966)		Pre-Birth Parents (*N* = 2342)		Post-Birth Parents (*N* = 3384)	
	Mean ± SD/%	*n*	Mean ± SD/%	*n*	Mean ± SD/%	*n*
Sex in %						
Female	44.9	8956	54.3	1272	55.4	1873
Male	55.1	11,010	45.7	1070	44.7	1511
Age in years (Mean, SD)	33.5 ± 10.6	19,966	28.7 ± 5.4	2342	34.8 ± 5.9	3384
Educational status in %						
Primary/secondary	69.9	13,964	48.8	1142	54.3	1838
Tertiary	30.1	6002	51.2	1200	45.7	1546
Partner status in %						
No partner	59.9	11,956	2.6	61	2.3	77
Partner	40.1	8010	97.4	2281	97.7	3307
Migration Background in %						
None	84.7	16,912	86.4	2023	86.1	2914
Direct or indirect	15.3	3054	13.6	319	13.9	470
Birth Cohort in %						
1960–1969	25.4	5065	3.0	69	3.1	106
1970–1979	20.2	4027	27.8	652	38.5	1304
1980–1991	54.5	10,874	69.2	1621	58.3	1974
Years of Observation in %						
2008	17.3	3444	29.3	687	0	0
2009–2010	19.7	3932	24.2	567	6.4	215
2011–2012	19.5	3885	22.8	535	12.6	426
2013–2014	14.6	2922	15.0	351	17.6	594
2015–2016	11.7	2.329	7.7	180	20.8	704
2017–2018	9.6	1912	0.9	22	23.0	778
2019	7.7	1542	0	0	19.7	667
HLI [0–8] (Mean, SD)	5.7 ± 1.6	11,322	6.0 ± 1.5	1923	5.8 ± 1.5	1110

Source: Own compilation and depiction using SOEP data (v.36), *N* = 25,692 person-years, imputed (*m* = 20 imputations).

## Data Availability

SOEP data can be obtained via DIW upon reasonable request.

## Abbreviations

The following abbreviations are used in this manuscript:

ATT	Average treatment effect on the treated;
BMI	Body Mass Index;
BY+1–3a	More than one to three years after birth;
BY+3–5a	More than three to five years after birth;
BY+5a+	More than five years after birth;
BY−/+1a	One year before to one year after birth;
BY−1-3	More than one to three years before birth;
BY−3a+	More than three years before birth;
CASMIN	Comparative Analysis of Mobility in Industrial Nations;
DIW	German Institute for Economic Research;
EVS III	Healthy Lifestyle Questionnaire;
GDM	Gestational Diabetes Mellitus;
GLQ	General Lifestyle Questionnaire;
HLI	Health Lifestyle Index;
HPLP II	Health-Promoting Lifestyle Profile II;
LE8	Life’s Essential 8;
LS7	Life’s Simple 7;
MEDLIFE	Mediterranean Lifestyle Index;
MI	Multiple imputation;
POLS-GT	Pooled ordinary least squares general trends;
Ref.	Reference category;
SES	Socioeconomic status;
SOEP	Socio-Economic Panel.

## Appendix A

**Table A1:** Commonly Used Health Lifestyle Indices.

Index Name	Items	Dimensions	Categorization of Behaviors	Index Construction
Healthy Lifestyle Questionnaire (EVS III) [82]	35	Balanced diet Respecting mealtimes Smoking Alcohol consumption	Likert scale from 1 (strongly disagree) to 5 (strongly disagree)	Not mentioned
Life’s Simple 7 (LS7) [83,84]	7	Smoking BMI Physical activity Diet Blood pressure Total cholesterol Blood glucose	0 (unhealthy) to 2 (healthy)	Sum score, values from 0 (unhealthiest) to 14 (healthiest)
Life’s Essential 8 (LE8) [85]	8	Diet Physical activity Nicotine exposure Sleep health BMI Blood lipids Blood glucose Blood pressure	0 (unhealthy) to 100 (healthy)	Unweighted average of all items, values from 0 (unhealthiest) to 100 (healthiest)
Mediterranean Lifestyle Index (MEDLIFE) [86]	28	Food consumption Traditional Mediterranean dietary habits Physical activity Rest habits Social interaction habits	Dichotomized, 0 (unhealthy) or 1 (healthy)	Sum score, values from 0 (unhealthiest) to 28 (healthiest)
General Lifestyle Questionnaire (GLQ) [87]	46	Cognitive activities Physical activity Social activities Other leisure activities Sleep Diet Alcohol consumption Smoking	Likert scale from 1 (never) to 5 (every day or almost every day)	Unweighted average of all items or for each dimension, values from 1 (unhealthy) to 5 (healthy)
Healthy Lifestyle Assessment Toolkit [88]	34	Anthropometric measurements Physical activity Well-being, social cohesion, functional independence Nutrition Mental health Smoking, drinking, illicit substance use Sleep habits and quality Health and disease	Low risk, moderate risk, relevant risk	For each dimension: low risk (if all items are low risk), moderate risk (if at least one item is moderate risk and none is relevant), relevant risk (if at least one risk is relevant)
Health-Promoting Lifestyle Profile II (HPLP II) [47,89]	52	Health responsibility Physical activity Nutrition Spiritual growth Interpersonal relations Stress management	Likert scale from 1 (never) to 4 (routinely)	Unweighted average of all items, values from 1 (unhealthiest) to 4 (healthiest)
Non-validated variations of a Health Lifestyle Index		Typically include: Diet Physical activity Smoking Alcohol consumption BMI [48,90,91,92] Less often: Sleep Sedentary behavior Cognitive activities [93,94,95]	Common categorizations: Dichotomized, 0 (unhealthy) or 1 (healthy) based on official recommendations or sample distribution [92,93] More nuances between unhealthy and healthy, based on official recommendations or sample distribution [96,97]	Common construction methods: Sum score [48,90,92,98] Weighted sum score for outcome-specific HLIs [95,98,99]

Source: Own depiction and compilation based on the literature cited in the table. Results were retrieved via a non-systematic literature search using the search terms “health lifestyle index” and “health lifestyle score” across MEDLINE, Web of Science, and Google Scholar.

## Appendix B

**Table A2:** Categorization of Variables.

Variable	Item	Answer Options	Final Categorization	
**Dependent Variables**				
Physical Activity	Which of the following activities do you take part in during your free time: Doing sports (2009, 2011, 2015, 2017)	Every week	Regularly	(2) Regularly (1) Irregularly (0) Never
		Every month	Irregularly	
		Less frequently		
		Never	Never	
	Now some questions about your leisure time. Please indicate how often you take part in each activity: Taking part in sports (2008, 2013)	Every day	Regularly	
		Every week		
		Every month	Irregularly	
		Less frequently		
		Never	Never	
	Now some questions about your leisure time. Please indicate how often you take part in each activity: Taking part in sports (2018, 2019)	Daily	Regularly	
		At least once a week		
		At least once a month	Irregularly	
		Rarer		
		Never	Never	
Diet	How much attention do you pay to maintaining a healthy diet?	Very strong		(2) Strong
		Strong		
		A little		(1) A little
		Not at all		(0) Not at all
Smoking	Do you currently smoke, whether cigarettes, a pipe, or cigars?	No	Nonsmoker	(2) Nonsmoker (1) Light smoker (0) Heavy smoker
		Yes	Smoker	
	How many cigarettes, pipes or cigars do you smoke per day?	X cigarettes per day	Light smoker: <20 cigarettes Heavy smoker: ≥20 cigarettes	
		X pipes per day	Light smoker: <7 pipes Heavy smoker: ≥7 pipes	
		X cigars per day	Light smoker: <5 cigars Heavy smoker: ≥5 cigars	
Sleep	How many hours do you sleep on average on a normal day during the working week?	(metric)	Sufficient: 6–9 h Insufficient: <6 h or >9 h	(2) Sufficient and satisfied (1) Insufficient or unsatisfied (0) Insufficient and unsatisfied
	How satisfied are you right now with the following areas of your life? Sleep:	0–10 Satisfaction	Satisfied: 6–10 Unsatisfied: 0–5	
HLI	Physical Activity	(0) not healthy (1) moderately healthy (2) healthy	Sum of scores	(0–8), metric
	Diet			
	Smoking			
	Sleep			
**Independent Variables**				
Time-varying parenthood	Year of birth 1st child	(metric)	Birth of 1st child in calendar time	(0) Interview before 1st birth (1) Interview after 1st birth
	Month of birth 1st child			
	Survey year		Interview in calendar time	
	Month of interview			
Time-constant parenthood	Time-varying parenthood	(0) Interview before 1st birth	No (1) during observation period	(0) Never parent
		(1) Interview after 1st birth	At least one (1) during observation period	(1) Ever parent
Time-sensitive parenthood	Birth of first child in calendar time	(metric)	No parenthood observed	(0) BY-3a+
			Interview > 3 years before birth	
			Interview > 1–3 years before birth	(1) BY−1-3a
	Interview in calendar time		Interview 1 year before to 1 year after birth	(2) BY−/+1a
			Interview > 1–3 years after birth	(3) BY+1-3a
			Interview > 3–5 years after birth	(4) BY+3-5a
			Interview > 5 years after birth	(5) BY+5a+
**Control Variables**				
Age	Survey year	(metric)	Survey year-birth year	Age (metric)
	Year of birth			
Birth cohort	Year of birth	(metric)		(1) 1960–1969
				(2) 1970–1979
				(3) 1980–1991
Partner status at birth	Partner indicator	No partner		(0) No partner
		Spouse, registered partner		(1) Partner
		Partner		
		Probably spouse, registered partner		
		Probably partner		
		Not clear		(.)
Migration background	Migration background	No migration background		(0) None
		Direct migration background		(1) Direct or indirect
		Indirect migration background		
Education at first birth	CASMIN classification	(0) In school	Primary	(1) Primary/ secondary
		(1a) Inadequately completed		
		(1b) General elementary school		
		(1c) Basic vocational qualification		
		(2b) Intermediate general qualification	Secondary	
		(2a) Intermediate vocational		
		(2c_gen) General maturity certificate		
		(2c_voc) Vocational maturity certificate		
		(3a) Lower tertiary education	Tertiary	(2) Tertiary
		(3b) Higher tertiary education		
Wave	Survey year	2008–2019		2008 2009–2010 2011–2012 2013–2014 2015–2016 2017–2018 2019
Sex	Sex	Male		(1) Male
		Female		(2) Female
		Nonbinary		(.)

Source: Own compilation and depiction using SOEP data (v.36) [100].

## Appendix C. Regression Tables

**Table A3:** Effect of Parenthood on HLI and Health Behaviors over Time for Women.

	HLI	Physical Activity	Diet	Smoke	Sleep
**Time before/since birth year (Ref.: BY-3a+)**					
BY-1-3a	−0.075	−0.085 **	−0.024	−0.024	0.057 **
	(0.077)	(0.042)	(0.035)	(0.032)	(0.025)
BY-/+1a	−0.196 **	−0.216 ***	−0.027	0.141 ***	−0.095 ***
	(0.096)	(0.050)	(0.041)	(0.039)	(0.030)
BY+1-3a	−0.759 ***	−0.475 ***	−0.080*	0.034	−0.238 ***
	(0.116)	(0.058)	(0.045)	(0.045)	(0.040)
BY+3-5a	−0.651 ***	−0.451 ***	−0.040	0.018	−0.178 ***
	(0.142)	(0.072)	(0.057)	(0.050)	(0.045)
BY+5a+	−0.707 ***	−0.470 ***	−0.095	−0.030	−0.111 **
	(0.172)	(0.088)	(0.069)	(0.062)	(0.055)
**Age**	−0.012 *	−0.006	0.006 **	−0.009 ***	−0.004
	(0.007)	(0.004)	(0.002)	(0.003)	(0.003)
**Education (Ref.: Primary/Secondary)**					
Tertiary	0.818 ***	0.332 ***	0.154 ***	0.233 ***	0.099 ***
	(0.046)	(0.022)	(0.017)	(0.018)	(0.015)
**Partner (Ref.: No partner)**					
Partner	0.070	−0.027	0.036 *	0.019	0.042 **
	(0.054)	(0.025)	(0.019)	(0.022)	(0.018)
**Migration status (Ref.: None)**					
Direct or indirect	−0.193 ***	−0.163 ***	0.001	0.002	−0.033 *
	(0.060)	(0.030)	(0.020)	(0.024)	(0.020)
**Birth cohort (Ref.: 1960–1969)**					
1970–1979	−0.049	−0.042	0.000	−0.037	0.030
	(0.121)	(0.056)	(0.036)	(0.050)	(0.043)
1980–1991	0.036	0.029	0.027	−0.062	0.042
	(0.174)	(0.082)	(0.054)	(0.071)	(0.059)
**Wave (Ref.: 2008)**					
2009–2010	0.005	−0.070 ***	0.006	0.016	0.052 ***
	(0.033)	(0.018)	(0.015)	(0.014)	(0.013)
2011–2012	−0.229 ***	−0.075 ***	0.031 *	−0.227 ***	0.042 ***
	(0.047)	(0.023)	(0.019)	(0.023)	(0.015)
2013–2014	0.191 ***	0.092 ***	0.018	0.041 *	0.039 **
	(0.053)	(0.028)	(0.021)	(0.021)	(0.018)
2015–2016	0.146 **	0.028	0.012	0.065 **	0.041 *
	(0.068)	(0.033)	(0.030)	(0.026)	(0.023)
2017–2018	0.166 **	0.024	0.022	0.089 ***	0.031
	(0.081)	(0.040)	(0.034)	(0.031)	(0.027)
2019	0.201 **	0.156 ***	0.035	0.005	0.006
	(0.098)	(0.046)	(0.042)	(0.042)	(0.032)
**Ever parent (GT)**	−0.937 ***	−0.452 ***	−0.053	−0.360 ***	−0.072
	(0.297)	(0.151)	(0.096)	(0.109)	(0.095)
**Ever parent X Age**	0.038 ***	0.019 ***	0.004	0.013 ***	0.001
	(0.011)	(0.006)	(0.003)	(0.004)	(0.003)
**Constant**	6.225 ***	1.451 ***	1.179 ***	1.853 ***	1.742 ***
	(0.325)	(0.155)	(0.105)	(0.130)	(0.110)
**Observations**	12,101	12,101	12,101	12,101	12,101

Source: Own compilation and depiction using SOEP data (v.36), *N* = 25,692 person-years. The pooled OLS group trend models regressed the individual health behavior variables stratified by gender on the covariates of age, educational level, relationship status, migration status, survey year, and birth cohort while integrating an interaction term between the parenthood indicator and age. Coefficients with robust standard errors in brackets show the difference in the proportion of HLI and single health-related indicators between parents and the same individuals, if they had (counterfactually) remained childless. Ref.: Reference category. * *p* < 0.10, ** *p* < 0.05, and *** *p* < 0.01.

**Table A4:** Effect of Parenthood on HLI and Health Behaviors over Time for Men.

	HLI	Physical Activity	Diet	Smoke	Sleep
**Time before/since birth year (Ref.: BY-3a+)**					
BY-1-3a	−0.021	−0.039	−0.015	0.010	0.023
	(0.085)	(0.043)	(0.039)	(0.041)	(0.026)
BY-/+1a	−0.023	−0.083 *	−0.024	0.088 *	−0.004
	(0.107)	(0.049)	(0.046)	(0.051)	(0.031)
BY+1-3a	−0.245 **	−0.246 ***	−0.043	0.098 *	−0.054
	(0.118)	(0.056)	(0.052)	(0.055)	(0.036)
BY+3-5a	−0.170	−0.255 ***	0.009	0.143 **	−0.067
	(0.142)	(0.067)	(0.065)	(0.064)	(0.044)
BY+5a+	−0.307 *	−0.278 ***	−0.061	0.100	−0.068
	(0.185)	(0.082)	(0.083)	(0.078)	(0.049)
**Age**	−0.034 ***	−0.019 ***	0.004*	−0.016 ***	−0.004 *
	(0.007)	(0.003)	(0.003)	(0.003)	(0.002)
**Education (Ref.: Primary/Secondary)**					
Tertiary	0.996 ***	0.363 ***	0.201 ***	0.336 ***	0.096 ***
	(0.048)	(0.023)	(0.018)	(0.020)	(0.014)
**Partner (Ref.: No partner)**					
Partner	0.120 **	0.005	0.049 **	0.014	0.052 ***
	(0.056)	(0.027)	(0.021)	(0.025)	(0.017)
**Migration status (Ref.: None)**					
Direct or indirect	0.007	0.019	0.060 ***	−0.023	−0.050 ***
	(0.063)	(0.028)	(0.022)	(0.027)	(0.019)
**Birth cohort (Ref.: 1960–1969)**					
1970–1979	−0.105	−0.083	−0.013	−0.108 **	0.099 ***
	(0.109)	(0.052)	(0.035)	(0.049)	(0.031)
1980–1991	−0.152	−0.076	−0.003	−0.161 **	0.088 *
	(0.163)	(0.078)	(0.058)	(0.075)	(0.049)
**Wave (Ref.: 2008)**					
2009–2010	0.007	−0.049 ***	0.020	−0.008	0.044 ***
	(0.032)	(0.016)	(0.016)	(0.015)	(0.012)
2011–2012	−0.231 ***	−0.048**	0.068 ***	−0.279 ***	0.027*
	(0.045)	(0.022)	(0.019)	(0.025)	(0.014)
2013–2014	0.207 ***	0.100 ***	0.033	0.029	0.045 ***
	(0.054)	(0.026)	(0.022)	(0.024)	(0.017)
2015–2016	0.193 ***	0.045	0.022	0.087 ***	0.038 *
	(0.067)	(0.033)	(0.031)	(0.030)	(0.021)
2017–2018	0.312 ***	0.077 **	0.037	0.166 ***	0.032
	(0.082)	(0.039)	(0.037)	(0.036)	(0.025)
2019	0.235 **	0.206 ***	0.058	−0.003	−0.026
	(0.100)	(0.045)	(0.043)	(0.049)	(0.030)
**Ever parent (GT)**	−0.864 ***	−0.194	−0.177	−0.314 **	−0.179 **
	(0.298)	(0.147)	(0.108)	(0.127)	(0.080)
**Ever parent X Age**	0.029 ***	0.009 *	0.006 *	0.009 **	0.005 **
	(0.010)	(0.005)	(0.003)	(0.004)	(0.002)
**Constant**	6.521 ***	1.833 ***	0.952 ***	2.000 ***	1.736 ***
	(0.313)	(0.149)	(0.111)	(0.145)	(0.092)
**Observations**	13,591	13,591	13,591	13,591	13,591

Source: Own compilation and depiction using SOEP data (v.36), *N* = 25,692 person-years. The pooled OLS group trend models regressed the individual health behavior variables stratified by gender on the covariates of age, educational level, relationship status, migration status, survey year, and birth cohort while integrating an interaction term between the parenthood indicator and age. Coefficients with robust standard errors in brackets show the difference in the proportion of HLI and single health-related indicators between parents and the same individuals, if they had (counterfactually) remained childless. Ref.: Reference category. * *p* < 0.10, ** *p* < 0.05, and *** *p* < 0.01.
